# Medical Training Initiative (MTI) fellowship – a personal reflection

**DOI:** 10.1192/bji.2019.38

**Published:** 2020-05

**Authors:** Halilu Abdullahi, Jonathan Wilson

**Affiliations:** 1 Early Intervention Services, Lancashire and South Cumbria NHS Foundation Trust, UK. Email: onlinehal@gmail.com; 2 Consultant Psychiatrist, Central Norfolk Youth Service, UK; 3 Research Director, Norfolk and Suffolk NHS Foundation Trust, UK; 4 Clinical Senior Lecturer, University of East Anglia, UK; 5 Professor of Psychiatry, St. George's University School of Medicine, Grenada

**Keywords:** MTI, Nigeria, fellowship, culture

## Abstract

Dr Abdullahi was a Fellow of the Royal College of Psychiatrists’ Medical Training Initiative (MTI) scheme at Norfolk and Suffolk NHS Foundation Trust, East Anglia, between 2015 and 2017. He has adopted a comparative approach to share some salient observations and anecdotes from that time.

The Medical Training Initiative (MTI) is a scheme sponsored by the Royal College of Psychiatrists (RCPsych) and designed to enable a small number of international psychiatrists enhance their skills and experience while training in the National Health Service (NHS) for up to 2 years. The expectation is that the MTI fellowship experience will in turn benefit their institutions, patients and colleagues upon their return to their home countries. I was a Fellow of the RCPsych MTI scheme at Norfolk and Suffolk NHS Foundation Trust in the East of England between 2015 and 2017, under the supervision of Dr Jon Wilson. In addition to the scheme expectations, I was hopeful of completing the RCPsych membership examinations, having previously trained and practiced briefly as a consultant psychiatrist at Federal Psychiatric Calabar, Nigeria.

I used to ponder back in Calabar, about how patients lacking in insight could make meaningful contributions to their treatment plans. To my surprise, the client-centred approach that was largely in vogue during the entirety of my MTI placement made me appreciate that even very ill patients (including those in in-patient settings) could still make rational and informed decisions regarding their care. This contrasts with the largely paternalistic approach in managing mental health problems in Nigeria, where key management decisions are usually made by attending psychiatrists or the patient's senior family members. I specifically remember observing colleagues at the Addictions Centre in Norwich ‘supporting’ patients who were not keen to reduce or cease their misuse of illicit substances. There were a few patients who regularly misused opiates on top of the ample doses of methadone conveniently prescribed by their psychiatrists over many years, and the professionals were quick to say ‘it is the patient's decision to make’. This would definitely be unusual practice in Calabar, as patients are generally and directly advised to desist from the substance misuse behaviour, which they tend to feel shameful about.

Nigerian patients, who are mostly from poor backgrounds and sometimes have to spend significant amounts of time and money travelling long distances, generally tend to attend for their appointments. As such, I was quite surprised to observe that in a climate of relative abundance, UK patients were prone to non-attendance and last minute cancellations, resulting in rescheduling of appointments. Although I am mindful that this observed pattern may be the result of a complex interaction of several factors, it brought to my mind parts of a long-forgotten elementary school rhyme, ‘some have food but cannot eat, some can eat but have no food’.

Furthermore, attitudes at the time of discharge are worth mentioning. For the quintessential patient in the Nigerian context, the time of discharge is usually a time of positive reflection, professed hope and most patients are quick to embrace the need to ‘move on’ from a difficult experience and stigmatising environment. This appears to be in contrast with the observed reluctance from some patients in UK community and in-patient mental health settings when discharge is being planned. In this context, I am reminded of a cheerful patient who became teary when discharge was mentioned and suddenly remembered that he had forgot to report a suicidal idea he had a month beforehand. I was subsequently able to realise that for some patients, support from mental health professionals may represent their only meaningful social contact, whereas some other patients seemingly prefer to keep certain secondary gains of well-supported sick roles – hence the reluctance to be discharged.

When patients come to mental health settings in the Nigerian context, they usually tend to be accompanied by family members, guardians or friends, which perhaps is an exemplification of well-known collectivistic attitudes, with family and community compensating for the inadequacies in social welfare support, among other possibilities. A fall-out of such attitudes is that psychiatric assessments are overwhelmingly reliant on the historical details provided by family and friends, consequently resulting in less time being spent interacting with patients. I also remember trying to resist asking about the whereabouts of parents of lonely adolescents mostly presenting all by themselves in my MTI placement at the Youth Service in Norwich. I was subsequently able to find my answers in their family backgrounds laden with abusive and chaotic childhood histories, weak or non-existent family bonds, attachment issues and periods of stay in foster homes, among other predisposing factors.

The specialist youth service where I started my MTI placement, which offers an intensive outreach model of treatment to young people (14–25 years) with complex comorbid emotional, behavioural and social problems, is non-existent in the Nigerian context where only the ‘privileged’ (defined here as being able to afford private healthcare) adolescent would usually be referred to a child or adult psychiatrist.^1^

The youth service in the UK forms part of a prevalent ‘functional’ model of care in which different psychiatrists are responsible for in- and out-patient care. I observed the functional (split) model to be perceived to reduce consultant stress, empower other professionals and give patients a chance to have a second opinion about their management when they move between functional teams. The alternative ‘sectorised’ model of care whereby the same psychiatrist is responsible for care across different settings is generally the case in Nigerian government-run psychiatric hospitals. I was able to note that continuity of care, therapeutic alliance, the doctor–patient relationship and trust are potentially better preserved with the sectorised model.

Suicidal behaviour and self-harm are generally abhorred in the Nigerian context, and the aftermath of such acts is fraught with disapproval and stigma from family and health professionals, although recently attitudes are starting to change. As such, and in line with extant research findings, most cases of self-harm and suicidal behaviour presenting to mental health services occur almost entirely in the context of major or severe mood, psychotic or substance-related disorders.^2^ I remember a mother who lashed her daughter (with a broom) for attempting to end her life following school examination difficulties. This observation differs considerably from the frequent and pervasive thoughts and acts of self-harm and suicide in persons with relatively less severe mental health diagnoses or social problems, and yet were well supported by health professionals during my fellowship. During my subsequent on-calls, I struggled somewhat with having to frequently assess patients in emergency settings or acute psychiatric wards who were not psychotic, depressed or under the influence of substances making constant threats of suicide. I also observed colleagues in liaison psychiatric environments being overburdened with crisis-stricken, emotionally unstable patients who would be largely dismissed as ‘badly behaved’ should they present in one of the Nigerian Federal neuropsychiatric hospitals. This made me ponder to what extent cultural attitudes and values contribute to the expression of these behaviours.

I was able to observe (using myself as a reference point) the majority of psychiatrists I worked with (some of whom were my supervisors) during my fellowship to be less rigid, less procrustean and generally reluctant to embrace case definitions when making psychiatric diagnoses. They are thus more likely to diagnose mental disorders outside extant classification schemes. As such, a person who is reactively sad for a few days or weeks in Nigeria would easily pass as mild or moderately depressed in the UK. Many cases of autistic disorder in Norfolk, for example, would easily pass as minor social or communication issues back in Calabar, Nigeria. These observations bring to mind the question of whether psychiatric symptomatology and diagnoses are valid across cultures.

UK newcomer psychiatrists should also be aware of the ‘idioms of distress’, frequently used by British patients, which tend to have well-recognised meanings and may be easily misinterpreted. I remember a manic patient mention that he was ‘full of beans’ and I replied ‘but you said you haven't been eating much’ in my misunderstanding!

I wrote, on completion of the RCPsych examinations, that ‘international psychiatrists who are not adept at the nuances of British verbal and non-verbal communication or are inadequate in their understanding of UK-specific mental health terminology would be at a disadvantage in their attempt at the Clinical Assessment of Skills and Competences (CASC) – the final stage of the MRCPsych examinations’. Although my training in Nigeria largely prepared me for the MTI experience, I suspect that prospective Nigerian MTI trainees will be a little less driven and find issues relating to appraisals, portfolio assessment, revalidation and administrative paperwork in the UK somewhat daunting (at least initially).

## Conclusion

The MTI fellowship provided me with an ‘eye-opening’ and first-hand experience of the contrasts that exist in resourcing, patient attitudes, psychopathological patterns, trainee assessments and the practice of psychiatry in very distinct settings.

I am also more aware of the role sociocultural and organisational factors play in the expression and management of mental disorders. The fellowship itself was quite fulfilling and getting to accomplish a post-nominal (MRCPsych) recognition in UK psychiatry in that time was the icing on the cake.
